# Supplementary material for: Empirical methods for controlling false positives and estimating confidence in ChIP-Seq peaks
Source: BMC Bioinformatics. 2008 Dec 5;9:523. doi: 10.1186/1471-2105-9-523 (PMC2628906; doi:10.1186/1471-2105-9-523)
Supplement: Additional File 1 — A variety of html documents from the USeq web site detailing the available applications, their best usage, output file type descriptions, command line menus, etc. [file 1471-2105-9-523-S1.zip › USeqUserGuides/JavaDoc.zip!/JavaDoc/bioroot/GeneBean.html]

GeneBean


|  |  |  |
| --- | --- | --- |
| **Overview** \| **Package** \| **Class** \| **Use** \| **Tree** \| **Deprecated** \| **Index** \| **Help** | |  |
| **PREV CLASS**   **NEXT CLASS** | **FRAMES**    **NO FRAMES**     **All Classes** |  |
| SUMMARY: NESTED \| FIELD \| CONSTR \| METHOD | DETAIL: FIELD \| CONSTR \| METHOD |  |


---


## bioroot Class GeneBean

```
java.lang.Object
  bioroot.GeneBean
```

---

``` public class GeneBean extends java.lang.Object ```

**Author:**
:   nix
    General class to hold information about an gene, used by Oligo, Plasmid, and Strain DB's.

---

**Constructor Summary**

`GeneBean()`

`GeneBean(int id,
DBUtil bioRoot)`


| **Method Summary** | |
| --- | --- |
| `java.lang.String` | `fetchHTMLTableRow()` |
| `java.lang.String` | `getEditHistory()` |
| `int` | `getId()` |
| `int` | `getLabGroupId()` |
| `java.lang.String` | `getMessages()` |
| `java.lang.String` | `getName()` |
| `java.lang.String` | `getNotes()` |
| `boolean` | `isComplete()` |
| `void` | `setComplete(boolean b)` |
| `void` | `setEditHistory(java.lang.String string)` |
| `void` | `setId(int i)` |
| `void` | `setMessages(java.lang.String string)` |
| `void` | `setName(java.lang.String string)` |
| `void` | `setNotes(java.lang.String string)` |
| `boolean` | `submitNew(DBUtil bioRoot, UserBean user)` |
| `boolean` | `updateOld(DBUtil bioRoot, UserBean user)` |

**Methods inherited from class java.lang.Object**

`equals, getClass, hashCode, notify, notifyAll, toString, wait, wait, wait`

**Constructor Detail**

### GeneBean

```
public GeneBean()
```

---


### GeneBean

```
public GeneBean(int id,
                DBUtil bioRoot)
```


**Method Detail**

### fetchHTMLTableRow

```
public java.lang.String fetchHTMLTableRow()
```

---


### submitNew

```
public boolean submitNew(DBUtil bioRoot,
                         UserBean user)
```

---


### updateOld

```
public boolean updateOld(DBUtil bioRoot,
                         UserBean user)
```

---


### getEditHistory

```
public java.lang.String getEditHistory()
```

---


### getId

```
public int getId()
```

---


### getMessages

```
public java.lang.String getMessages()
```

---


### getName

```
public java.lang.String getName()
```

---


### getNotes

```
public java.lang.String getNotes()
```

---


### setEditHistory

```
public void setEditHistory(java.lang.String string)
```

---


### setId

```
public void setId(int i)
```

---


### setMessages

```
public void setMessages(java.lang.String string)
```

---


### setName

```
public void setName(java.lang.String string)
```

---


### setNotes

```
public void setNotes(java.lang.String string)
```

---


### isComplete

```
public boolean isComplete()
```

---


### setComplete

```
public void setComplete(boolean b)
```

---


### getLabGroupId

```
public int getLabGroupId()
```


---


|  |  |  |
| --- | --- | --- |
| **Overview** \| **Package** \| **Class** \| **Use** \| **Tree** \| **Deprecated** \| **Index** \| **Help** | |  |
| **PREV CLASS**   **NEXT CLASS** | **FRAMES**    **NO FRAMES**     **All Classes** |  |
| SUMMARY: NESTED \| FIELD \| CONSTR \| METHOD | DETAIL: FIELD \| CONSTR \| METHOD |  |


---
